# Chromatin accessibility and differentially expressed genes profiling in large yellow croaker (*Larimichthys crocea*) head kidney cells following iridovirus infection

**DOI:** 10.3389/fimmu.2025.1513966

**Published:** 2025-01-30

**Authors:** Chaowei Song, Ying Huang, Fang Han, Zhiyong Wang

**Affiliations:** ^1^ State Key Laboratory of Mariculture Breeding, Key Laboratory of Healthy Mariculture for the East China Sea, Fujian Provincial Key Laboratory of Marine Fishery Resources and Eco-Environment, Fisheries College, Jimei University, Xiamen, China; ^2^ Laboratory for Marine Fisheries Science and Food Production Processes, Qingdao Marine Science and Technology Center, Qingdao, Shandong, China

**Keywords:** chromatin accessibility, iridovirus infection, epigenetics, immune gene expression, ATAC-seq

## Abstract

**Introduction:**

The large yellow croaker iridovirus (LYCIV) poses a significant threat to the aquaculture industry of *Larimichthys crocea*. Understanding the host defense response to LYCIV infection is crucial for developing effective strategies to mitigate its impact.

**Methods:**

In this study, an epigenetic approach was employed to investigate dynamic changes in chromatin accessibility using the assay for transposase-accessible chromatin with high-throughput sequencing (ATAC-seq). Additionally, RNA sequencing (RNA-seq) was used to analyze the expression pattern of immune response genes upon LYCIV infection.

**Results:**

Substantial alterations in chromatin accessibility were observed, particularly in the regulatory regions of key immune-related genes. Significant changes in the expression of AP-1 transcription factors, including the *Batf* gene, were noted. CUT&Tag results revealed that AP-1 was significantly enriched in the open chromatin regions of cytokine genes, with *Batf* potentially regulating the cytokine genes *LIF* and *CLCF1*.

**Discussion:**

These findings suggest that AP-1 may play a crucial role in the defense response against viral infection by modulating inflammatory cytokines and contributing to cellular inflammatory responses. This study provides a comprehensive analysis of the epigenomic landscape and gene expression regulation during iridovirus infection in *L. crocea*, offering valuable insights for breeding programs aimed at combating iridovirus infections.

## 1 Introduction

The large yellow croaker (*Larimichthys crocea*) with its distinctive golden yellow body color is of great economic importance in marine fisheries in China. According to the 2023 China Fisheries Statistical Yearbook, nationwide aquaculture production of large yellow croaker reached 257,700 tons in 2022, making it the leading marine aquaculture species (1). The iridovirus disease is a prevalent viral infection in large yellow croaker. Iridovirus is an enveloped dsDNA virus of the *Iridoviridae* family, with a genome size of about 112 kb (2, 3). According to the International Committee on Taxonomy of Viruses (https://ictv.global/taxonomy), iridoviruses are currently classified into seven genera, namely *Iridovirus*, *Megalocytivirus*, *Ranavirus*, *Lymphocystivirus*, *Chloriridovirus*, *Decapodiridovirus*, and *Daphniairidovirus* (4). Among these, *Megalocytivirus* represents one of the most significant pathogens responsible for high mortality in many finfish species (5). The genus *Megalocytivirus* comprises two species, *Megalocytivirus lates1* and *Megalocytivirus pagrus1*. The large yellow croaker iridovirus (LYCIV) belongs to the *Megalocytivirus pagrus1* and is closely related to red sea bream iridovirus (RSIV), infectious spleen and kidney necrosis virus (ISKNV), turbot reddish body iridovirus (TRBIV) and rock bream iridovirus (RBIV) (6), all of which are part of the *Megalocytivirus* genus (4). The infection normally manifests as body darkening, reduced appetite, and severe tissue damage in organs including the liver, spleen, and kidneys, with mortality rates reaching up to 75% (7). Understanding the defense response of large yellow croaker against iridovirus attack, especially the induced-gene expression profile, is crucial for advancing knowledge in virology and immunology (8).

An interesting topic of epigenetic regulation of gene expression is the impact of chromatin structure and organization. The structural state of chromatin directly influences the interactions between transcription factors and the genome, facilitating the access of transcription machinery (9). In other words, the chromatin accessibility is a pivotal switch for the potential onset of regulated gene expression, with the open chromatin region favoring the initiation of regulatory activities (10). The accessibility of these open chromatin regions is not static but dynamic (11). This dynamic nature allows genes within the genome to appropriately respond to external stimuli, facilitating processes such as cell differentiation and defense responses (12).

Assay for Transposase Accessible Chromatin with high-throughput sequencing (ATAC-seq) is a powerful tool to probe chromatin accessibility at whole genome level (13). It has been applied to investigate changes in host chromatin accessibility following pathogen infection and their effects on immune responses (14). The integration of ATAC-seq and RNA-seq identified candidate transcription factors (TFs) involved in viral responses in rice infected with RSV (15). The comparison of chromatin accessibility in phagocytic versus non-phagocytic cells in the head kidney of grass carp revealed that increased phagocytic capacity was correlated with reduced chromatin accessibility at promoter regions, which affected the accessibility of the transcription factors Irf4 and Irf8 crucial for B cell development (16–18). Moreover, analysis of macrophage chromatin state in obesity model mice versus control mice identified ETV5 as a critical transcription factor (19). These applications indicate a great potential of ATAC-seq as a useful technique to uncover the host immune regulatory network by epigenetic modulation.

Viral infections can significantly increase the chromatin accessibility of host antiviral regulatory elements, thereby enhancing the expression of antiviral genes. The scaffold protein SAFA (hnRNPU) can regulate the opening and closing of antiviral regulatory elements to regulate the expression of antiviral immune genes upon recognition of viral double-stranded RNA presence (20). Disruption of SAFA expression in cells notably reduces the chromatin accessibility of virus-inducible genes, while the accessibility of non-virus-responsive genes remains largely unaffected (21). On the other hand, some DNA virus infections can change the location and organization of host chromatin leading to an extensive remodeling to facilitate the viral replication (11). Investigation of host chromatin structural changes in accessibility across the genome in response to viral infection is important for a better understanding of host-virus interaction and host immune response. Currently, there is limited research on chromatin accessibility changes in fish following viral infection, highlighting the necessity of further exploration in this field.

In this study, the ATAC-seq technology was employed to study the dynamic changes in chromatin accessibility in large yellow croakers following iridovirus infection, and the coupled RNA-seq investigation was performed to explore how these changes influence the expression of host immune genes. Analysis of ATAC-seq and RNA-seq data on large yellow croaker cells at various time points of iridovirus infection revealed that the viral infection significantly altered chromatin openness, particularly in the regulatory regions of key immune-related genes. These findings offer new insights into the intricate antiviral activities and contribute to the development of novel antiviral strategies.

## 2 Materials and methods

### 2.1 Experimental fish collection and virus identification

About 100 juvenile large yellow croakers with typical symptoms of iridovirus infection were collected from an aquaculture center (Ningde City, Fujian Province), for the preparation of a virus infection solution. Diseased fish were identified by observing the characteristics of diseased fish (body darkening, reduced appetite, and severe tissue damage in organs including the liver, spleen, and kidney) and using specific PCR primers. These symptomatic fish had an average body length of 9.57 ± 2.43 cm, a body height of 2.52 ± 0.48 cm, and a body weight of 11.25 ± 1.75 g. The fish harvested were immediately frozen in liquid nitrogen and stored at -80°C to maintain sample integrity. The sample collection and experimental procedures were approved by the Jimei University Animal Care and Use Committee and strictly adhered to the “Regulations for the Administration of Affairs Concerning Experimental Animals” issued by the State Council of China. Virus suspension preparation was performed as described in previous studies as following (3). To prepare the virus infection solution, the liver, spleen, and kidneys of the diseased fish after thawing were harvested in sterile 2 mL microcentrifuge tubes and homogenized on ice using a tissue homogenizer (60 Hz, 120 s). After dilution in serum-free DMEM (1:10 w/v) supplemented with double antibiotics (penicillin-streptomycin) and amphotericin B, the homogenized samples were incubated in a water bath at 15°C for 2-4 h or at 6°C for 6-24 h. The samples were then centrifuged at 4°C at 5,000 rpm for 5 min. The obtained supernatant was filtered through a 0.22 µm filter and the filtrate containing virus was stored at -80°C.

Viral DNA was extracted using a specialized viral DNA extraction kit (ER201-01, TransGen). PCR amplification was performed on the extracted DNA using LYCIV-specific primer pairs LYCIV-F1 and LYCIV-R1 (**Table 1**) targeting the major capsid protein (MCP) gene (GenBank OK149102.1), and LYCIV-F2 and LYCIV-R2 (**Table 1**) targeting the ATPase gene (GenBank KY774434.1), respectively. The amplicon was examined by agarose gel electrophoresis.

**Table 1:** List of iridovirus identification primers, virus load detection primers, and RT-qPCR analysis primers.

Primer name	Sequence (5’-3’)	Application
LYCIV-F1	CTCAAACACTCTGGCTCATC	Virus identification
LYCIV-R1	GCACCAACACATCTCCTATC	
LYCIV-F2	ATTTGAATGCCAGCCTGAGG	
LYCIV-R2	TGTGCACTTGCTTACACCAC	
LYCIV-F3	GCCTACATGGACCACCTC	Virus copy number determination
LYCIV-R3	CGACCCTGCTACTTCTTC	
*interleukin-1β*-F	CGTGCTGGAGAACATAGTGGAAGAG	RT-qPCR analysis
*interleukin-1β*-R	TCCGCTGTCTTTGTGTACTGAACTG	
*irf8*-F	AGAATCTTCGTCGCCTCCATTGTG	
*irf8*-R	GCCGATGGGTTTGCTCTCTGTC	
*il13ral*-*like*-F	ATAAGACGCCTGACGAAACACTGAC	
*il13ral*-*like*-R	GCAAATGATAGCCCTGTACCTCCTG	
*ccl19*-F	CCACCATCATCAGATCACTGGACAG	
*ccl19*-R	GCAGGCACGCACAGTTTAATCTTAC	
*fgf21*-F	TGTCCTCACACCACGGATCTCC	
*fgf21*-R	CACACTCTCCCTCGCCAAAGTATTC	
*g-lysozyme*-F	GCCCAGCGAGAGGACAATACAAC	
*g-lysozyme* -R	CAGTAGCTTGGCAGAGGTGTTCC	
*pim1*-F	GAACACGACGAGGAGATCACAAGAG	
*pim1*-R	CCGCAGAGACAGACACCACTTG	
*rpa1*-F	TCTCCAGCCTTAACCCATACCAGTC	
*rpa1*-R	TCTCCACTCTCGTCCACAACCTC	
*cdk6*-F	CTTTGGTCTAGCCCGCATCTACAG	
*cdk6*-R	CTGGTGTGGCGTAACTGGACTG	
*β-actin*-F	TTATGAAGGCTATGCCCTGCC	
*β-actin*-R	TGAAGGAGTAGCCACGCTCTGT	

### 2.2 Viral load quantification and cell culture

A 197-bp PCR amplicon by the primer pair of LYCIV-F3/R3 (**Table 1**) targeting the iridovirus ATPase gene (**Supplementary Figure S1**) was constructed into the pEASY-T1 cloning vector. The recombinant plasmid was transformed into Trans1-T1 competent cells for replication. After isolation and quantification, a serial 10-fold dilution of the recombinant plasmid was prepared. A viral load standard curve (R²= 0.9981) was established using the serial dilutions as the standards by real time quantitative PCR (RT-qPCR) (**Supplementary Figure S2**), which was used as a reference for iridovirus viral load determination based on the cycle threshold.

The large yellow croaker head kidney (LYCK) cell line (22) was cultured in Leibovitz’s L-15 medium supplemented with 10% fetal bovine serum at 28°C. Cells in optimal growth conditions were digested with trypsin and seeded into 6-well plates. Once the cells reached 80% confluence, they were infected with iridovirus using an infection solution with a concentration of 5×10^4^ copies/µL and a multiplicity of infection (MOI) of 100. The cultures were then maintained at 28°C and sampled at 0, 12, 24, and 36 hours post infection (hpi), respectively, with three replicates at each time point. After centrifugation to remove trypsin, the cells were resuspended in 10 μL of PBS for subsequent library construction for ATAC-seq or RNA-seq.

### 2.3 ATAC-seq library preparation and data analysis

After determining the total and viable cell count in the cell resuspension using a Luna II cell counter, approximately 50,000 viable cells were harvested by centrifugation at 800 g for 5 min. The cells were resuspended in 50 μL of precooled lysis buffer containing 10 mM Tris-HCl (pH 7.4), 10 mM NaCl, 3 mM MgCl_2_, and 0.1% IGEPAL CA-630, and lysed on ice for 10 min to release the nuclei. The nuclei were collected by centrifugation at 500 g for 5 min in 4°C and subjected to a transposition reaction and library construction using the TD501 ATAC-seq Kit (Vazyme, China). After the transposition reaction, DNA was captured and purified using pre-equilibrated magnetic beads, followed by two washes with 80% ethanol. Library fragment size distribution was ensured to match the expected mononucleosome or dinucleosome model using Qseq-100 (Bioptic, China). The total concentration of the library was estimated using Qubit 3.0 fluorometer (Life Technologies, US), and high-throughput sequencing was performed on Illumina NovaSeq platform with a 150 bp paired-end mode (23).

After data trimming and quality control using Trim-galore (v2.10) (the parameters are set to -q 25 –phred33 –length 35 -e 0.1 –stringency 4 –paired) (24), and FastQC (v0.11.9) (25), the clean reads were aligned to the reference genome of *L. crocea* (https://www.ncbi.nlm.nih.gov/assembly/GCF_000972845.2#) using Bowtie2 v7.5.0 (26), the parameters are set to -p 5 –very-sensitive -X 2,000. The duplicate reads, mitochondrial reads, and low-quality alignment reads were filtered out using Sambamba v0.7.1 to obtain high-quality alignments (27) using default parameters. The enrichment of transcriptional initiation sites (TSS) was identified using DeepTools v3.4.2 (28). Peaks were identified using Macs2 v2.2.7.1 (29) (-g 7.5e08 –nomodel –shift -100 –extsize 200). The consensus peaks from three replicates of each stage were determined using Bedtools v2.29.2 (30). Annotation of genomic locations of the ATAC-seq peaks was achieved using ChIPseeker v1.24.0 package (31). Significantly enriched motifs in peaks were identified using the tool of Homer v4.10 (32), with motifs of 8, 10, and 12 in length.

To analyze open chromatin regions, the Diffbind package (33) was used to calculate read counts for each peak, adjust peak intervals between samples, centralize and prune peaks based on read overlaps, and evaluate differences based on edgeR with a significance threshold of FDR < 0.05 (34). The 5 kb in front of TSS was defined as the potential promoter region, and the position of peaks near each gene was distinguished, such as intron region, distal intergenic region, and so on. An online bioinformatics tool jvenn (https://www.bioinformatics.com.cn/static/others/jvenn/example.html) was used to obtain significant enrichment motifs intersections of peaks at each infection point, and those common motifs were identified.

### 2.4 RNA-seq library construction and data analysis

Total RNA was extracted using TRIzol reagent (Invitrogen, Carlsbad, CA, United States), and its quality was assessed by agarose gel electrophoresis and further analyzed using a Nanodrop spectrophotometer and a Qubit fluorometer and Agilent 2100 bioanalyzer. The library was created using TIANSeq Stranded RNA-Seq Kit (Illumina) and subjected to the quality control analysis using Qubit fluorometer and Agilent 2100 bioanalyzer. Once the inserted fragment size met our expectations, the library was quantified using qPCR. The libraries were then pooled and sequenced on the Illumina Novaseq 6000 sequencing platform.

The RNA-seq data was cleaned using STAR v2.7.6a (35) to align clean reads to the reference genome (https://www.ncbi.nlm.nih.gov/assembly/GCF_000972845.2#), and the transcripts were assembled using StringTie v2.1.2 (36). Transcript abundance was calculated using featureCounts v1.0 (37), and differentially expressed genes (DEGs) were identified using DESeq2 v1.28.1 (38). The criteria for defining differentially were |log2(fold change)| > 1 and adjusted *p-*value < 0.05. The Gene Ontology (GO) enrichment analysis of the DEGs within each group was performed using clusterProfiler v3.16.1 (39). The AnnotationHub Server (v2.20.2) (40) was used to obtain GO annotation information for the large yellow croaker. The Kyoto Encyclopedia of Genes and Genomes (KEGG) pathway enrichment was analyzed with DOSE v3.14.0 (41), with a *p-*value < 0.05 for significant enrichment.

SYBR Green-based RT-qPCR was performed to analyze the expression of *interleuin-1beta*, *irf8*, *il13ra1-like*, and *ccl19* (genes involved in the cytokine-cytokine receptor interaction pathway) using the 2^−ΔΔCT^ method. RT-qPCR was also performed to analyze the expression of *fgf21*, *g-lysozyme*, *pim1*, *rpa1*, sbspon and *cdk6* (genes involved in processes such as cellular stress, DNA damage response, and immune regulation) using the 2^−ΔΔCT^ method. *β-actin* was chosen as the reference gene for normalization (see primers in **Table 1**).

### 2.5 *Batf* gene expression analysis

An overexpression vector was designed based on the CDS sequence of the *batf* gene (NCBI: XM_010735250.3), and primers were designed and synthesized by Sangon Biotech (Shanghai, China). The single-stranded primers were annealed into double-stranded Oligo sequences and connected to the double-enzyme linearized overexpression vector (pBHGlox(delta)E13Cre). The correct transformants were verified by sequencing, and high-purity plasmid extraction was performed. Thus, a plasmid containing the Ad-shuttlescr-*batf* overexpression vector was obtained. The adenovirus shuttle plasmid carrying the exogenous gene and the auxiliary packaging plasmid carrying most of the adenovirus genome were co-transfected into HEK293 cells, and the recombination was achieved using the Cre/loxP recombinase system to produce recombinant adenovirus (38, 39). The HEK293T and LYCK cells were transfected with a negative control adenovirus vector and those transfected with the *Batf*-overexpression adenoviral vector. RT-qPCR was performed to analyze the induced expression of *batf* gene with *β-actin* as the internal control (**Table 1**). Western blot was performed following the standard procedures using anti-Flag as the primary antibody.

### 2.6 Adenovirus infection and CUT&Tag library construction

LYCK cells were detached and diluted to 3×10^5^ cells/mL. Subsequently, 1.5×10^5^ cells (500 μL/well) were seeded into each well of a 24-well plate and incubated overnight at 28°C. Adenovirus was thawed on ice and adjusted to viral titer of 1.07×10^11^ PFU/mL at the time of infection. Based on preliminary experiments, an MOI (multiplicity of infection) of 100 was deemed suitable. After 4 h of infection, 500 μL of complete culture medium was added. Following an additional 6-8 h of incubation, the virus-containing culture medium was removed, replaced with fresh complete medium, and cells were further cultured at 28°C.

The cells were detached using trypsin digestion. Once completely dissociated from the culture surface, the digestion was halted using complete culture medium. The cells were then mixed, centrifuged at 800 g for 5 min. After removing the supernatant, 1 mL of PBS was added, and a 1:1 mixture of 10 μL of cell suspension and Trypan blue was prepared, and the cells were counted using a cell counter with three biological replicates. The cultured cells were then washed and bound to ConA beads. The cell-ConA bead complex was then co-incubated with the primary antibody, followed by incubation with the secondary antibody. Hyperactive pA/G-Transposon was added for TTBL tagmentation. The DNA was then extracted, and the library was amplified and purified. The distribution and concentration of library fragments were detected (39, 40).

The parts containing Adapter, N, or low-quality bases in the raw sequencing data were filtered out by Cutadapt v4.8.137 (42) to obtain high-quality sequencing data (clean reads) for follow-up alignment. Bowtie2 v7.5.0125 (26) was used to align the high-quality sequencing data with the reference genome (https://www.ncbi.nlm.nih.gov/assembly/GCF_000972845.2#), and then the results were evaluated for better quality. After removing reads from the mitochondrial sequence, the CUT&Tag signal (peaks) was scanned across the entire gene range using peak-calling software Macs2 v2.2.7.1 (29). The position information of peaks in the genome and the Peaks length information were obtained. Bedtools v2.29.2 (30) was used to merge two biologically consistent Peaks.

### 2.7 Data availability

The datasets [ATAC-seq, RNA-seq, CUT&Tag] generated in this study can be found in the [PRJNA820167], and [PRJNA1116991] of Sequence Read Archive (SRA).

## 3 Results

### 3.1 Identification of iridovirus and cytopathic effects

**Figure 1:**
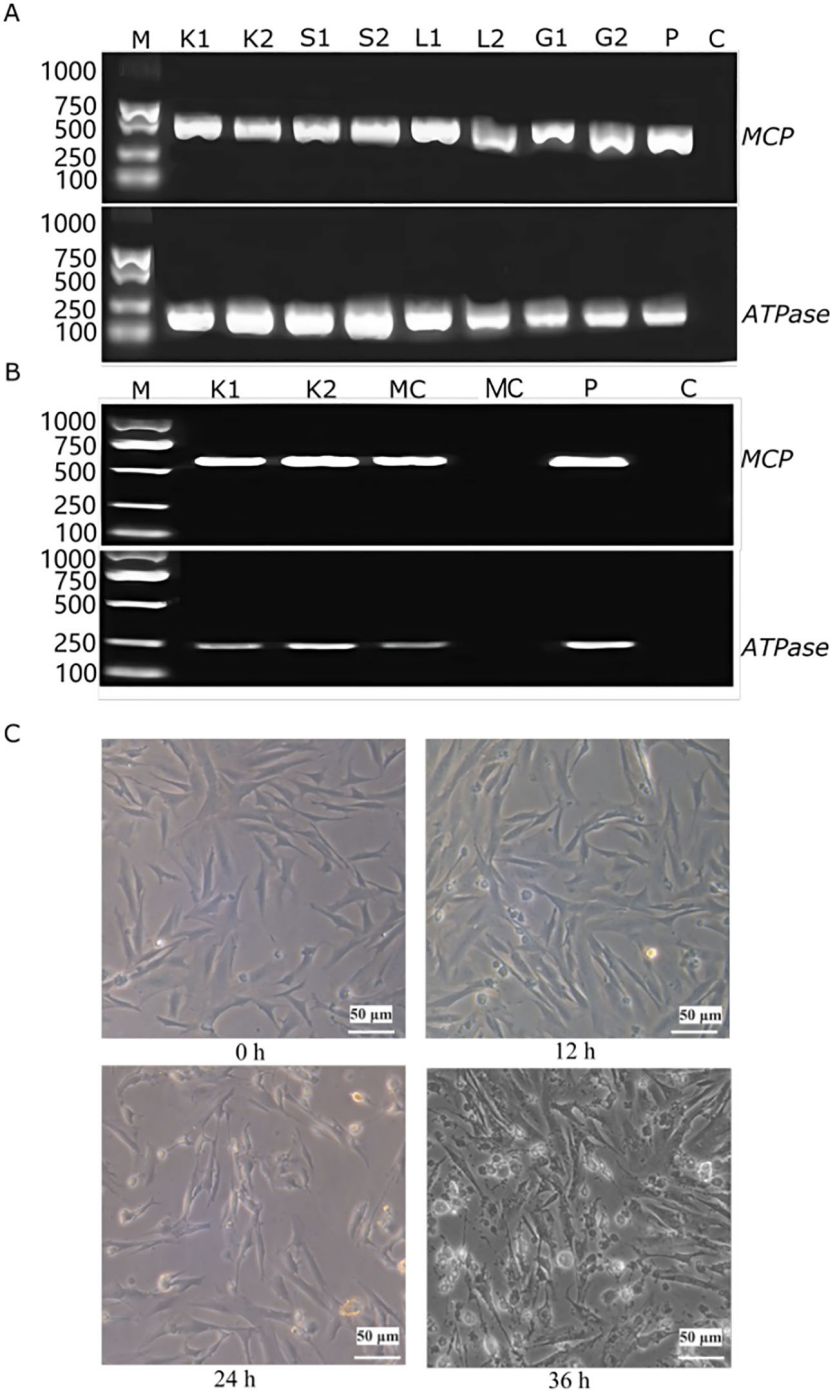
PCR identification of LYCIV and its cytopathic effect in LYCK cells. **(A)** Identification of LYCIV in diseased fish by PCR. The fragments of MCP gene and ATPase gene of LYCIV were detected by their specific primers. K1 and K2 are for kidney, S1 and S2 for spleen, L1 and L2 for liver, and G1 and G2 for gill of two biological replicates, respectively. P stands for positive control and C for blank control. **(B)** Identification of LYCIV in infected LYCK cells by PCR. MC stands for culture medium. The others are the same as above. **(C)** Cytopathic effects in LYCK cells following LYCIV stimulation. Shows progressive changes at 0, 12, 24, and 36 h, including vacuolization and cellular contraction, highlighting LYCIV's impact on cell health.

The presence of large yellow croaker iridovirus (LYCIV) in various organs (liver, spleen, mid-kidney, gills) of the symptomatic large yellow croaker was confirmed by PCR. A specific band was detected in the diseased fish but was absent in the non-template control (**Figure 1A**). In addition, the LYCIV was specifically detected in the large yellow croaker cell line and the culture media post infection (**Figure 1B**), which indicated a successful colonization of LYCIV in the LYCK cells.

Phenotypically, the LYCK cells post infection exhibited noticeable vacuolization and other cytopathic effects (CPE), which was intensified over the course of the infection, including increased cellular vacuolization and contraction (**Figure 1C**).

### 3.2 Peak calling and genomic distribution of ATAC-seq peaks

**Figure 2:**
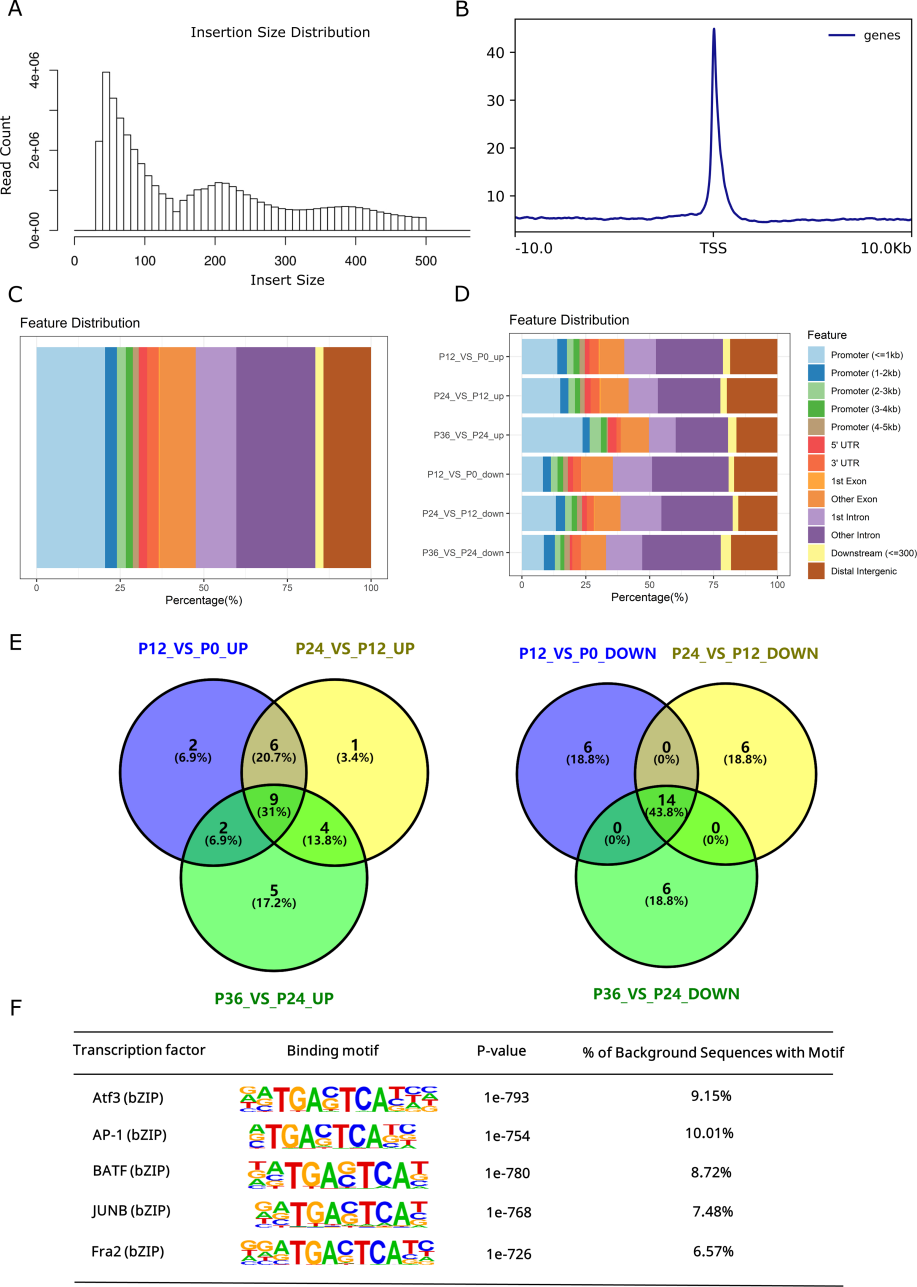
Genomic distribution of ATAC-seq Peaks and their sequence motifs analysis. **(A)** Length distribution map of the inserted fragment. The first peak represents the non-nucleosome model, the second peak represents the mono-nucleosome model, and the third peak represents the bi-nucleosome model. **(B)** Enrichment analysis of TSS. For the upstream and downstream 10 kb range of each gene TSS, the signal intensity of chromatin accessibility was calculated. The upstream and downstream 10 kb ranges of all gene TSS regions were combined and compared with random regions, and the enrichment degree of TSS region was determined by statistical analysis. **(C)** Genomic distribution of identified ATAC-seq peaks. Genome annotation includes promoter regions relative to the TSS site and other specified regions. **(D)** Proportion of significantly upregulated and downregulated peaks in different regions. P0, P12, P24, and P36 denote the chromatin accessibility at the time points of 0, 12, 24, and 36 hpi, respectively. Genome annotation includes promoter regions relative to the TSS site and other specified regions. **(E)** Venn diagrams of top 20 enriched motifs in regions of altered chromatic accessibility. **(F)** Common transcription factors enriched in open chromatin throughout the time course of infection.

The ATAC-seq library at each time point of infection was constructed and rigorous quality control was performed. After analysis of the ATAC-seq data, the clean reads, the alignment rate to the reference genome, and the enrichment at transcriptional starting sites (TSS) were listed (**Table 2**). The average size of the ATAC-seq clean data was 7.98 Gb, and the average alignment rate to the reference genome (GCF_000972845.2) was above 90% for each group, suggesting a good quality of the sample preparation. The length distribution of insert fragments showed three distinct nucleosome models, with peaks at approximately 190 bp, 350 bp, and 520 bp, respectively (**Figure 2A**). TSS enrichment analysis revealed significant enrichment of signals near transcription start sites (**Figure 2B**). Most of the ATAC-seq peaks were located in gene promoter regions, introns, and distal intergenic regions (**Figure 2C**).

**Table 2:** ATAC-seq data quality control at different time points.

Group	Read1	Read2	Alignment rate (%)	Frip (%)	Peak
0	5,616,522,267	5,592,487,382	94.99	69.64	82.406
0	4,043,341,206	4,026,663,085	89.12	58.80	84,791
0	4,507,440,253	4,486,996,209	89.63	56.28	84,804
12	4,341,930,192	4,327,518,013	91.40	67.24	84,377
12	3,536,553,032	3,537,394,596	92.02	62.32	86,008
12	3,818,220,508	3,817,867,189	94.80	62.18	90,050
24	2,849,171,109	2,840,225,825	88.07	53.24	79,656
24	4,414,283,115	4,402,388,228	90.97	43.45	82,200
24	4,476,645,874	4,464,157,009	91.65	59.33	88,636
36	4,193,297,981	4,181,175,951	94.18	62.04	85,719
36	3,821,960,508	3,812,497,409	92.64	53.61	87,091
36	3,761,337,004	3,763,017,970	92.51	57.55	82,630

### 3.3 Dynamic changes in chromatin accessibility and motif analysis

Systematical analysis of chromatin accessibility changes in LYCK cells in response to LYCIV infection showed that 12,201 regions exhibited decreased chromatin accessibility, while 8,884 regions exhibited increased accessibility at 12 hpi compared to the control (P12_VS_P0). Additionally, 16,167 regions had decreased and 16,228 regions had increased accessibility at 24 hpi compared to those at 12 hpi (P24_VS_P12). Furthermore, 325 regions showed decreased and 181 regions showed increased accessibility at 36 hpi compared to those at 24 hpi (P36_VS_P24) (**Table 3**). The dynamic changes of chromatin accessibility suggested an active chromatin remodeling in LYCK cells in response to the viral attack. The percentage of upregulated ATAC-seq peaks in the promoter regions increased consistently, while the percentage in distal intergenic regions gradually decreased (**Figure 2D**). The top 20 enriched motifs in regions with chromatin accessibility changes across the three comparisons (P12_VS_P0, P24_VS_P12, P36_VS_P24) were analyzed and showed in the Venn diagram (**Figure 2E**), and some motifs common to all three time points were identified such as AP-1 transcription factor motifs. The AP-1 family involved includes Batf, JunB, Atf3 and other transcription factors, which shared a core binding motif of TGACTCA (**Figure 2F**).

**Table 3:** The number of regions with significant up and down chromatin accessibility.

Group	Upregulation	Downregulation
P12_VS_P0	8884	12201
P24_VS_P12	16228	16167
P36_VS_P24	181	325

### 3.4 RNA-seq analysis and pattern of DEGs

**Figure 3:**
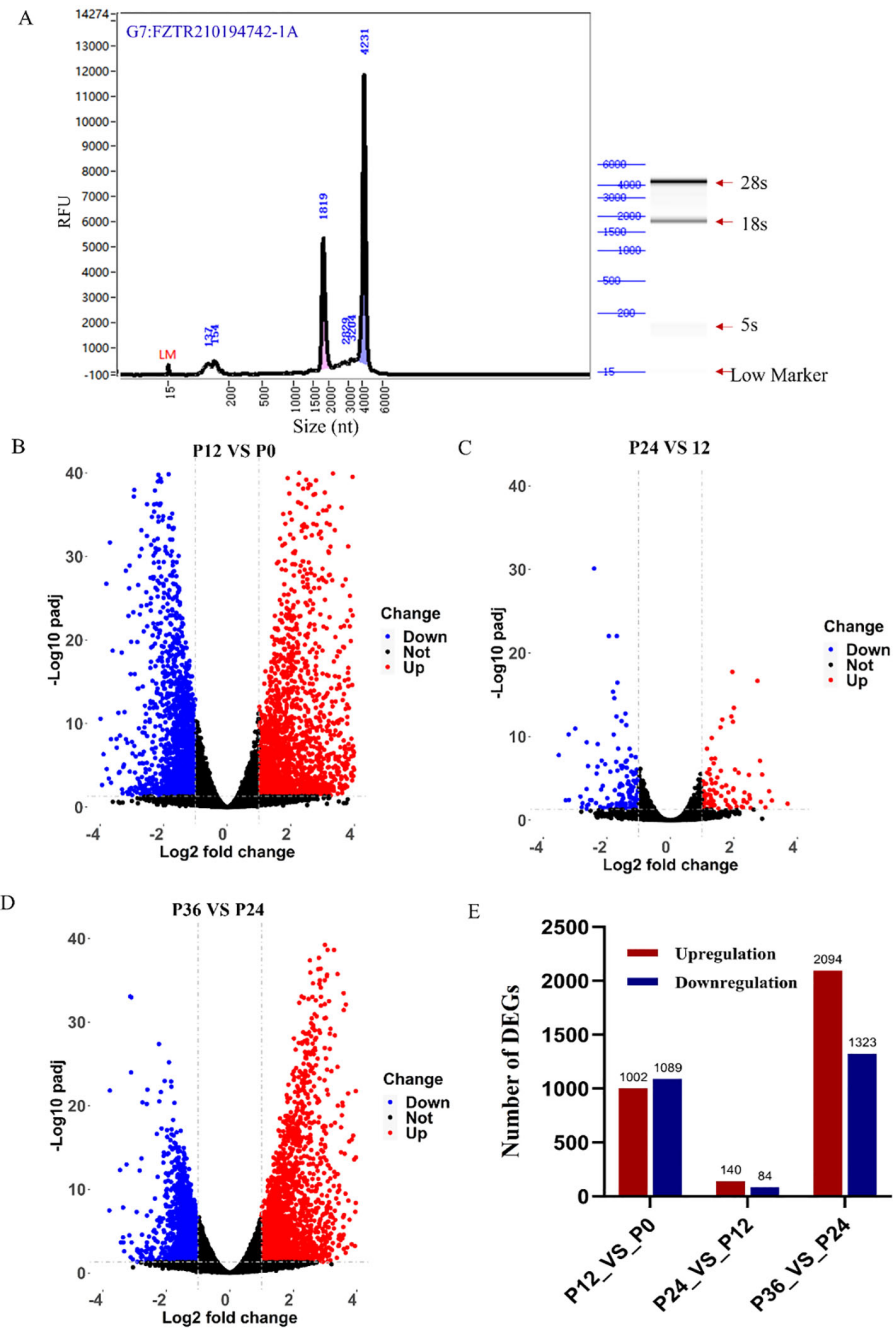
RNA-seq analysis of gene expression changes during LYCIV infection in LYCK cells. **(A)** Quality control of RNA-seq library. The quality of the library was analyzed with an Agilent bioanalyzer. **(B–D)** Illustration of the significant DEGs by Volcano plots in pairwise comparisons between P12 and P0 **(B)**, between P24 and P12 **(C)**, and between P36 and P24 **(D)**. Red dots indicate significantly up-regulated genes, blue ones indicate significantly down-regulated genes, and black ones indicate no significant differentially expressed genes. **(E)** The number of DEGs in the pairwise comparisons.

After RNA-seq library preparation, the quality of the library was assayed using Agilent bioanalyzer. Based on the strip sizes in the simulated gel chart, 28S, 18S, 5S, and Lower Marker bands were identified (**Figure 3A**). All 12 libraries passed quality control and met the sequencing requirements. RNA-seq of the 12 libraries produced a total of 85.22 Gb of data, with an average of approximately 7.10 Gb per library. After filtration, 81.85 Gb of data remained, with an average of about 6.82 Gb per library. The Q20 ratio ranged from 95.38% to 97.64%, the Q30 ratio from 90.00% to 93.86%, and the GC content from 44.46% to 47.77% (**Table 4**).

**Table 4:** Quality control for RNA-seq data.

Sample	Raw Reads	Clean Reads	Q20(%)	Q30(%)	GC Content (%)
D-0-1	42,853,314	41,703,624	95.38	90	44.46
D-0-2	46,721,022	45,584,260	95.58	90	44.71
D-0-3	44,243,154	43,183,822	97.42	93.17	46.02
D-12-1	51,720,548	49,720,488	96.78	92	47.75
D-12-2	55,236,824	52,139,714	97.64	93.84	47.77
D-12-3	45,497,130	43,181,294	97.54	93.43	46.45
D-24-1	44,921,032	44,267,774	97.40	93.15	46.66
D-24-2	45,184,104	44,095,852	97.23	92.80	46.58
D-24-3	45,437,282	42,676,176	97.28	92.85	45.73
D-36-1	48,877,698	46,754,400	97.73	93.86	46.58
D-36-2	53,057,506	50,755,470	96.66	91.77	46.77
D-36-3	44,278,840	41,612,408	97.32	92.91	45.66

In the P12_VS_P0 group, 1,089 genes were significantly downregulated, and 1,002 genes were significantly upregulated (**Figure 3B**). In the P24_VS_P12 group, 84 genes were significantly downregulated, and 140 genes were significantly upregulated (**Figure 3C**). In the P36_VS_P24 group, 1,323 genes were significantly downregulated, and 2,094 genes were significantly upregulated (**Figure 3D**). The number of differentially expressed genes at each time point was shown in the chart (**Figure 3E**).

### 3.5 GO and KEGG analyses of DEGs

**Figure 4:**
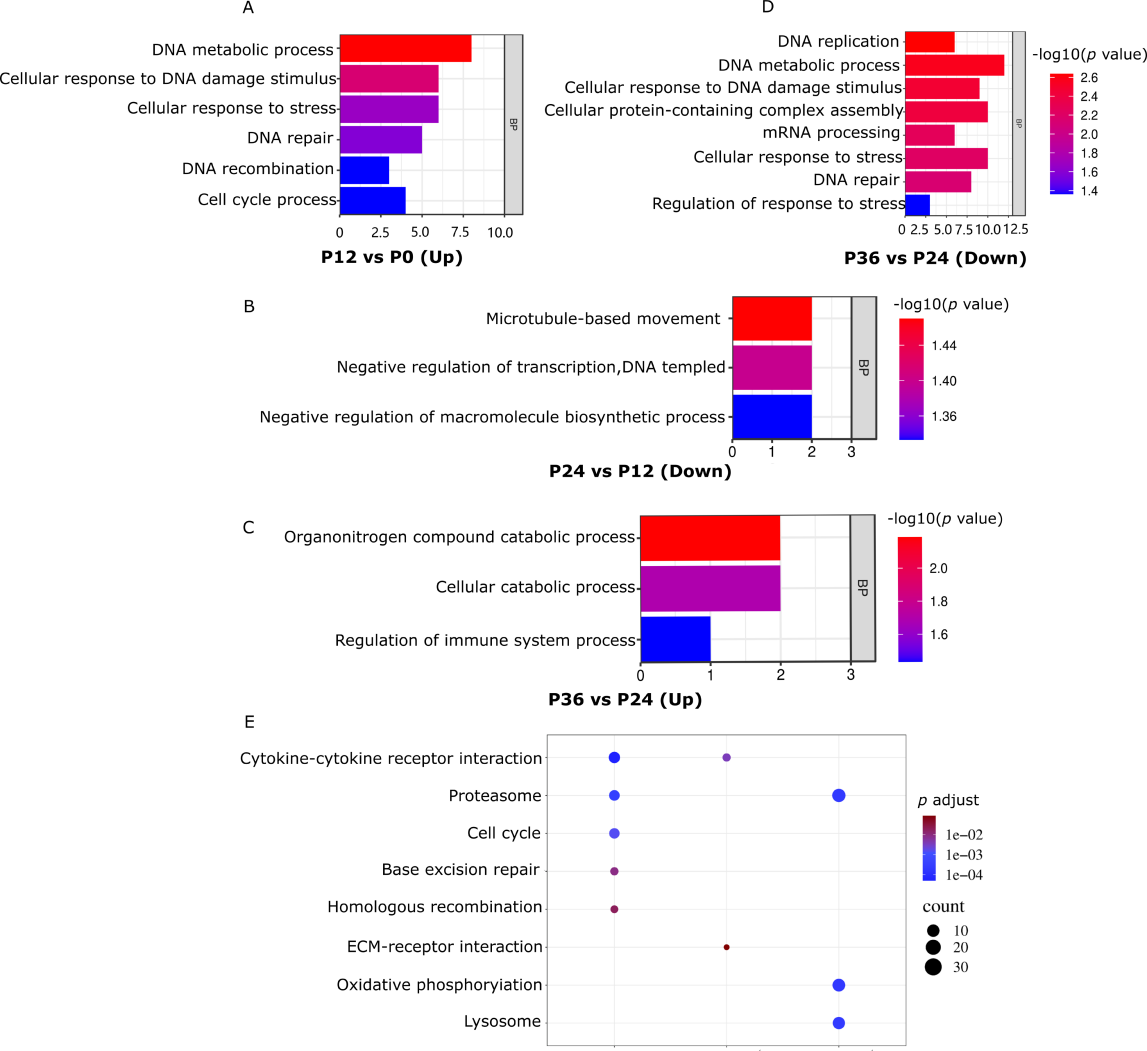
GO and KEGG analyses of differentially expressed genes during LYCIV infection progression. **(A–D)** Significantly enriched biological process GO categories of DEGs. **(E)** Significantly enriched KEGG pathways of upregulated and downregulated DEGs. The color indicates the significant adjust *p* value (*q* adjust) after correction in KEGG enrichment analysis, and the circle size indicates the number of genes enriched in this pathway.

Gene ontology (GO) analysis revealed that the upregulated gene set in P12_VS_P0 (P12_VS_P0_UP) was significantly enriched in pathways related to DNA damage response (**Figure 4A**). Concurrently, the downregulated gene set in P24_VS_P12 (P24_VS_P12_DOWN) showed significant enrichment in pathways associated with immune system regulation (**Figure 4B**). The upregulated gene set in P36_VS_P24 (P36_VS_P24_UP) was notably enriched in processes related to negative transcriptional regulation (**Figure 4C**). In contrast, the downregulated gene set in P36_VS_P24 (P36_VS_P24_DOWN) was significantly enriched in pathways related to DNA damage response, stress response processes, and pressure response regulation (**Figure 4D**).

KEGG analysis showed the gene sets in P12_VS_P0_UP and P24_VS_P12_DOWN was significantly enriched in the cytokine-cytokine receptor interaction pathway. Similarly, the gene set in P12_VS_P0_UP and the gene set in P36_VS_P24_DOWN were significantly enriched in the lysosome pathway (**Figure 4E**).

**Figure 5:**
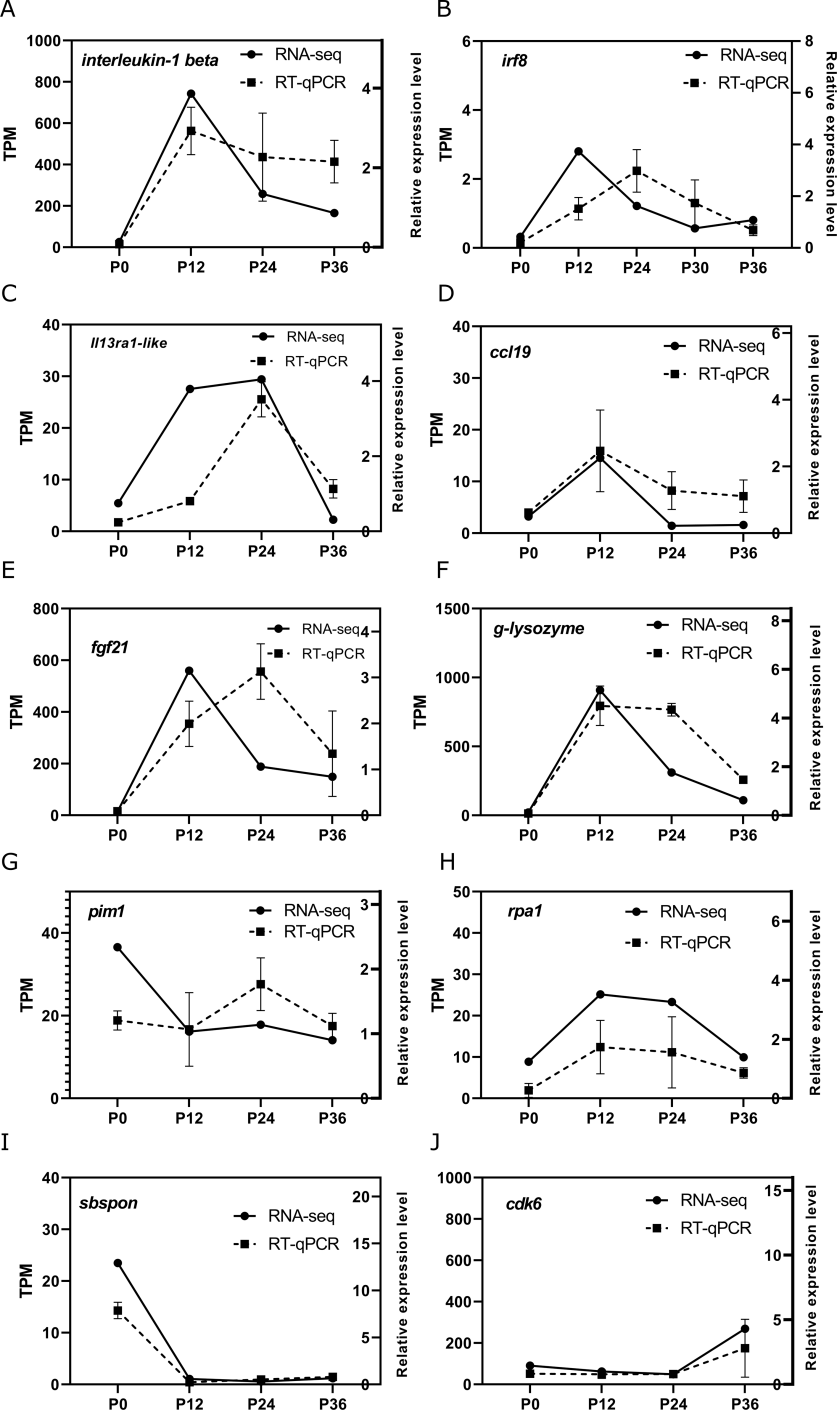
Validation of key differentially expressed genes (DEGs) related to various pathways by RT-qPCR. The temporal expression patterns of selected DEGs (**(A–J)**: *interleukin-1 beta, irf8, il13ra1-like, ccl19, fgf21, g-lysozyme, pim1, rpa1, sbspon, cdk6*) were analyzed at four time points (P0, P12, P24, and P36, corresponding to 0, 12, 24, and 36 h post-infection [hpi]) using RT-qPCR (broken line) and compared with the RNA-seq data (solid line). The left y-axis indicates the transcript abundance in transcripts per million (TPM) from RNA-seq analysis, while the right y-axis represents the relative expression levels of genes determined by RT-qPCR.

To validate the RNA-seq data, a set of genes were selected to examine their expression pattern by RT-qPCR, including the genes involved in cellular stress, DNA damage response, and immune regulation. The analysis of transcript abundance represented by TPM (transcripts per million) in RNA-seq and the relative expression level represented by normalized RT-qPCR showed a good correlation, indicating good reliability of the RNA-seq data (**Figures 5A–J**).

### 3.6 Synergistic changes in chromatin accessibility and gene expression

**Figure 6:**
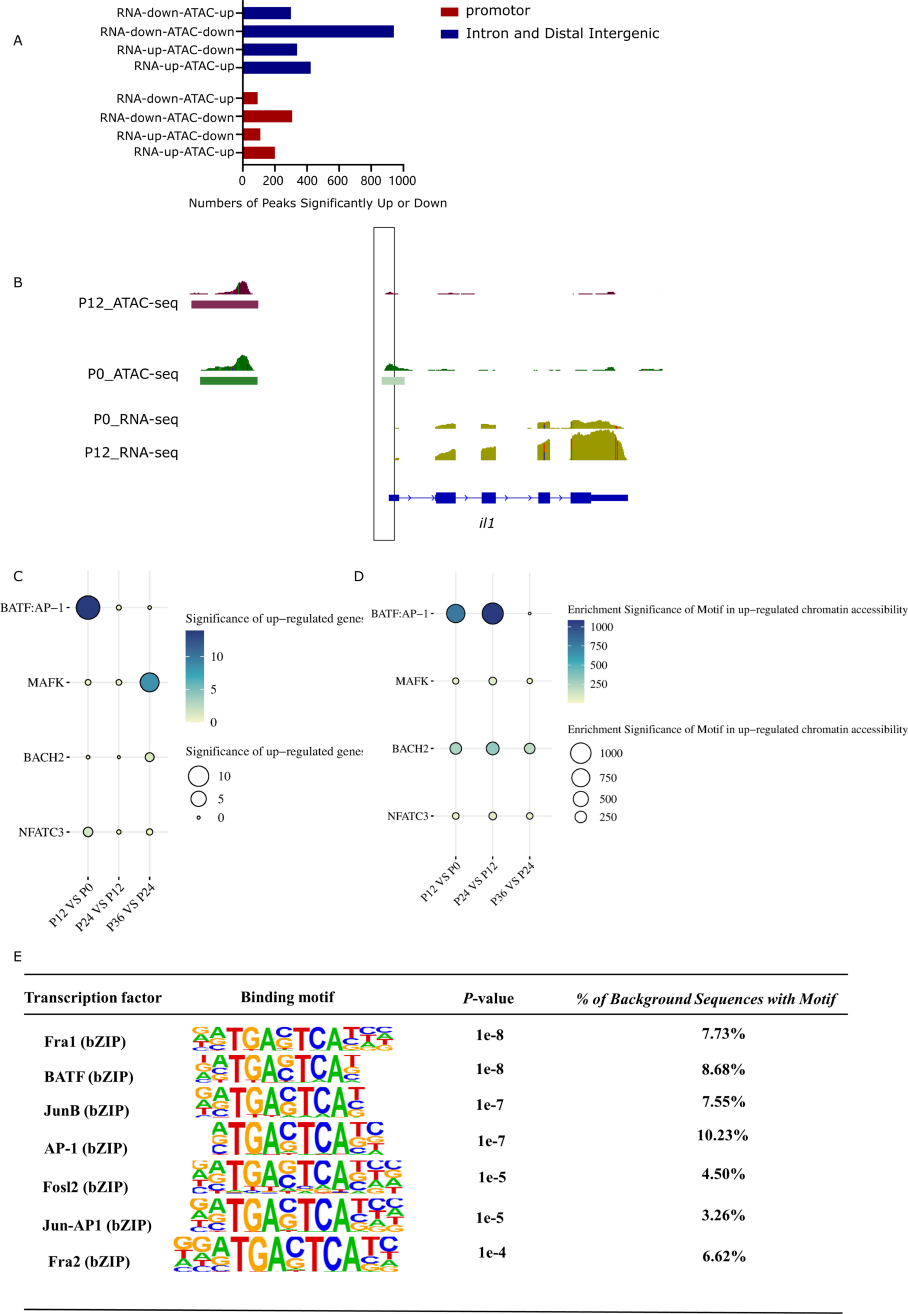
Correlation between chromatin accessibility and gene expression. **(A)** Quantitative relationship between changes in chromatin accessibility and gene expression in different non-coding regions. The X axis represents the number of genes, and the Y axis expresses the different combinations between chromatin accessibility and gene expression. **(B)** Change of chromatin accessibility in the noncoding region of the *IL1* gene. **(C)** Identification of key transcription factors in LYCK in response to LYCIV infection. The size of each circle and darker shades of blue and white correspond to the significance degree of up-regulated expression of transcription factors at each time points. Both color depth and size represent the up-regulated expression of transcription factors. The significance takes the negative logarithm of *p* value. **(D)** Identification of key transcription factors in LYCK responds to LYCIV infection. The size of each circle and darker shades of blue and white correspond to the significance of enrichment of transcription factor motifs with significantly up-regulated chromatin accessibility at each stage. Both color depth and size represent the significance of transcription factor enrichment. The significance takes the negative logarithm of *p* value. **(E)** Motifs significantly enriched in open chromatin regions of a variety of differentially expressed cytokine genes.

To further elucidate the dynamic changes in chromatin accessibility and gene expression during LYCIV infection in LYCK cells, the differential chromatin accessibility peaks from ATAC-seq and the DEGs from RNA-seq were compared with different combinations. The results indicated that the number of upregulated peaks was greater than that of downregulated peaks near upregulated genes, while the number of downregulated peaks near downregulated genes was greater than that of upregulated peaks, indicating a positive correlation between the chromatin accessibility and gene expression in a good amount of genome regions (**Figure 6A**). For example, the promoter region of the upregulated cytokine gene *il1* exhibited increased chromatin accessibility after 12 h of infection, as evidenced by a substantial increase in ATAC-seq peaks. Concurrently, the gene expression significantly increased, as indicated by the higher stacking height of RNA-seq reads on the transcript exon (**Figure 6B**).

Some key transcription factors that showed significant upregulation of gene expression and significant enrichment in chromatin accessible regions following infection were of great interest. Among the four common transcription factors (BATF: AP-1, MAFK, BACH2, and NFATC3), BATF, a member of the AP-1 transcription factor family, was significantly upregulated with a *p*-value < 0.01 in the comparison between P12 and P0 of RNA-seq data (**Figure 6C**). Simultaneously, there was significant enrichment of the BATF motif in the upregulated chromatin accessibility regions, with a *p*-value < 0.01 in the comparison between P12 and P0 (**Figure 6D**), further substantiating the relationship between chromatin state and regulatory gene expression.

From the non-coding regions of differentially expressed cytokine genes (promoter, intron, and intergenic regions), significantly enriched transcription factor binding motifs were extracted from the ATAC-seq peaks. The results showed that the TRE (TGAC/GTCA) regulatory element of AP-1 was significantly enriched in the open chromatin regions of these non-coding regions of cytokine genes (**Figure 6E**).

### 3.7 Target verification of CUT&Tag

**Figure 7:**
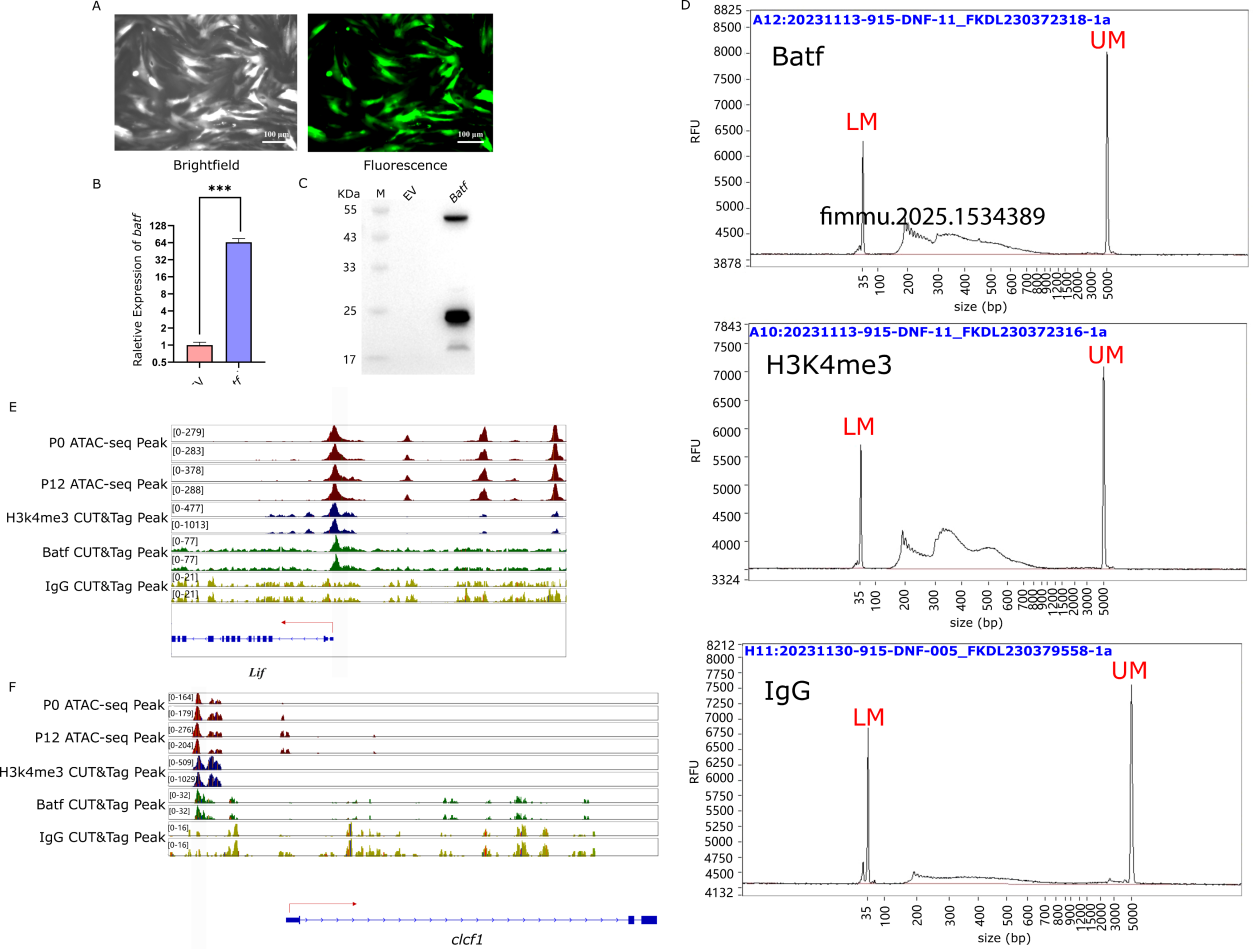
Target verification of CUT&Tag. **(A)** Efficiency of adenovirus transfection into LYCK. Images were captured at 48 h after infection. **(B)** Overexpression of Batf gene. The relative gene expression level was examined by RT-qPCR. The three-star symbol (*** ) indicates extremely significant differences, with *p* < 0.001. (EV stands for the empty vector of AD-shuttlescr). **(C)** Western blot analysis of Batf expression. (EV stands for the empty vector of AD-shuttlescr). **(D)** CUT&Tag library length distribution of Batf, H3K4me3, IgG antibodies. Abscissa indicates the fragment size of the library, and the ordinate (RFU) value refers to the real-time fluorescence signal strength and represents the sample concentration. LM (Lower Marker, 20bp) represents the minimum calibration marker, UM (Upper Marker, 5000bp) represents the maximum calibration internal parameter marker. **(E)** Batf binding signal in the open chromatin region of the promoter of cytokine *LIF* gene. The red lines represent the ATAC-seq signals of infected cells at 0 h and 12 h, the blue lines represent the H3K4me3 signals, the green lines represent the CUT&Tag signals of Batf, and the yellow lines represent the CUT&Tag signals of IgG. **(F)** Batf binding signal in the open chromatin region of the promoter of cytokine *CLCF1* gene. The red lines represent the ATAC-seq signals of infected cells at 0 h and 12 h, the blue lines represent the H3K4me3 signals, the green lines represent the CUT&Tag signals of Batf, and the yellow lines represent the CUT&Tag signals of IgG.

To verify whether the key transcription factor AP-1 regulates the expression of cytokine genes, *Batf* gene was cloned into an adenovirus vector with GFP expression cassette and transfected into LYCK cells. Fluorescence microscopy showed strong expression of *Batf-GFP* (**Figure 7A**), suggesting successful transformation and expression of *Batf* gene. In addition, the expression of *Batf* gene was examined by RT-qPCR and western blot. The RT-qPCR indicated abundant transcripts of *Batf* gene (**Figure 7B**), and the Flag-tagged Batf protein was detected specifically in the transfected cells with the *Batf* expression cassette (**Figure 7C**).

The antibody-guided CUT&TAG library preparation showed that the H3K4me3 library exhibited a regular pattern of three nucleosome models, indicating correct library preparation. In contrast, the experimental Batf and IgG libraries displayed irregular patterns, with the IgG library overall showing lower concentrations (**Figure 7D**). The offline data from the CUT&Tag sequencing were filtered and aligned to the reference genome of *L. crocea*, achieving an alignment rate of over 90%, with an average of 91.87%, which was sufficient for subsequent analysis (**Table 5**).

**Table 5:** CUT&Tag quality control data for Batf.

Group	Clean reads	Mapping rate (%)
H3K4me3-1	113,530,862	92.92
H3K4me3-2	214,294,438	92.63
IgG-1	31,757,390	86.17
IgG-2	43,200,932	92.63
Batf-1	107,537,546	91.16
Batf-2	103,640,562	91.06

The CUT&Tag assay results demonstrated that Batf bound to the open chromatin regions of the cytokine gene *LIF*. Peaks of H3K4me3 and open chromatin were observed in the promoter region of the *LIF* gene, along with Batf-targeted binding peaks, but no signal from IgG (**Figure 7E**). Similarly, Batf was bound to the open chromatin region of the cytokine gene *CLCF1* (**Figure 7F**). In the promoter region of the *CLCF1* gene, peaks of H3K4me3 and open chromatin were present, along with Batf-targeted binding peaks, and no signal from IgG. These findings were consistent with the results from previous ATAC-seq and RNA-seq analyses, further confirming the targeted regulation of cytokine genes by AP-1.

## 4 Discussion

The results of ATAC-seq revealed significant changes in chromatin accessibility within open chromatin regions during the progression of iridovirus infection. These changes likely reflect how host cells regulate gene expression at the chromatin level to adapt to iridovirus infection. Iridovirus infection induced significant alterations in chromatin architecture, leading to the reorganization of host gene expression patterns. This dynamic change in chromatin accessibility is comparable to the changes observed in host chromatin accessibility caused by Bombyx mori nucleopolyhedrovirus (BmNPV) infection in previous studies (43). We observed that iridovirus infection resulted in a notable increase in chromatin accessibility at several immune-related loci, particularly in non-coding regions regulating the cytokine-cytokine receptor interaction pathway. This is consistent with previous reports indicating that viral infections can induce changes in host chromatin structure to enhance the expression of immune genes (20). For instance, the scaffolding protein SAFA (hnRNPU) has been shown to recognize viral double-stranded RNA and selectively modulate the accessibility of antiviral regulatory elements, thereby boosting the expression of immune genes (20).

In our study, the significantly enriched GO and KEGG pathways of differentially expressed genes at various time points suggested a response pattern of LYCK cells to iridovirus infection. This view is supported by KEGG analysis and aligns with previously reported results of host defense failure caused by rock sea bream iridovirus (RBIV) (44).

We found that the interaction between cytokines and cytokine receptors is the most significantly enriched pathway in the KEGG analysis. However, one factor we cannot exclude is that regulatory elements responsible for upregulating chromatin accessibility might also be associated with the expression of non-cytokine genes. Additionally, it is possible that the upregulation of cytokine gene expression could be related to regions where chromatin accessibility remains unchanged nearby. To address these concerns, we conducted motif analysis on the open chromatin regions of all cytokine genes to identify upstream transcription factors regulating cytokine gene expression. This analysis revealed significant enrichment of AP-1 motifs in the open chromatin regions of non-coding regions associated with cytokine genes, suggesting a potential targeted regulatory role of the transcription factor AP-1 on cytokines.

ATAC-seq is highly sensitive in detecting potential transcription factor binding sites, offering valuable insights into the regulatory mechanisms activated in response to iridovirus infection (45). Our study identified AP-1 as a key transcription factor, with its binding sites showing increased chromatin accessibility following infection. Activator Protein-1 (AP-1) is crucial for various biological processes. It functions as a heterodimer composed of members from different DNA-binding protein families. AP-1 binds to TPA response elements (TRE) and cAMP response elements (CRE; TGAC/GTCA) (46). Its diverse functions are part of a complex and dynamic network of signaling pathways, which depend on the composition of its subunits and their interactions with other nuclear factors (47). In this study, we observed that the batf gene, a member of the AP-1 family (basic leucine zipper ATF-like transcription factor), was significantly upregulated 12 hpi and was notably enriched in regions of increased chromatin accessibility.

Nevertheless, further validation of AP-1’s targeted regulation is required. Conducting AP-1 ChIP-Seq experiments would provide more definitive evidence; however, performing ChIP-Seq in non-model species presents challenges such as high background noise, poor reproducibility, lack of highly specific antibodies, and the complexity of library construction (48). To overcome these limitations, our study employed adenoviral infection of LYCK cells to introduce an overexpression of Batf and take advantage of the tagged protein for CUT&Tag assay. In the western blot experiment involving the overexpression of Batf, a band near 55 kDa was observed, which may represent the dimer form of the Batf transcription factor. Previous studies have demonstrated that the leucine zipper domain of Batf facilitates dimerization with members of the Jun protein family (49). Additionally, some bands near 25 kDa were also observed in the western blot assay for Batf overexpression. These bands may be related to the phosphorylation of JNK kinase, which is associated with the activation of AP-1 transcriptional binding activity (50). AP-1 members frequently undergo post-translational modifications, such as phosphorylation by JNK kinase. The addition of phosphate groups increases the negative charge of the phosphorylated protein, leading to slower migration through the gel and an apparent increase in molecular weight, which was observed in c-Fos, another member of the AP-1 family (51).

In CUT&Tag experiment, the transposase cuts at the regions where the primary and secondary antibodies are bound. H3K4me3 (trimethylation of lysine 4 on histone H3) is typically located in open chromatin regions, so the transposase-antibody complex that binds to H3K4me3 will cut nearby nucleosomes, resulting in a characteristic nucleosome pattern in the library length distribution showing a regular pattern of three nucleosome models in a well-prepared library. On the other hand, IgG binds non-specifically to genomic regions and can be used to exclude non-specific binding regions in the experiment. Results from Batf-directed CUT&Tag analysis revealed Batf binding signals in the open chromatin regions of the promoters for cytokine genes *LIF* and *CLCF1*, suggesting that Batf may potentially regulate these cytokine genes. This finding is consistent with previously reported inflammatory responses mediated by Batf2/Irf in macrophages (52). Based on the results from ATAC-seq, RNA-seq, and CUT&Tag analyses, it is suggested that AP-1 may potentially regulate the expression of numerous cytokines and participate in the regulation of the inflammatory response. This is consistent with previously reported findings that AP-1 plays a role in regulating the expression of inflammatory cytokines (47, 53–55).

Our study provides a comprehensive epigenetic landscape of chromatin accessibility in large yellow croaker in response to iridovirus infection. The significant changes observed in chromatin openness at immune-related genes and the identification of key transcription factors involved in the host immune response offer valuable insights into the host’s antiviral response mechanisms. These findings provide novel perspectives on the complex mechanisms underlying host antiviral responses and could contribute to the development of new antiviral strategies.

## Data Availability

The names of the repository/repositories and accession number(s) can be found below: https://www.ncbi.nlm.nih.gov/; PRJNA1116991 and PRJNA820167 (SRA).

## References

[1] Wang D Wu F-X Song D-D Gao H-Q Liu X-Z Cui L-F. The China fishery statistical yearbook. 1st ed. Beijing: China Agriculture Press (2022).

[2] Wang X-W Ao J-Q Li Q-G Chen X-H. Quantitative detection of a marine fish iridovirus isolated from large yellow croaker, *Pseudosciaena crocea*, using a molecular beacon. J Virol Methods. (2006) 133:76–81. doi: 10.1016/j.jviromet.2005.10.025 16310867

[3] Chen XH Lin KB Wang XW. Outbreaks of an iridovirus disease in maricultured large yellow croaker, *Larimichthys crocea* (Richardson), in China. J Fish Dis. (2003) 26:615–9. doi: 10.1046/j.1365-2761.2003.00494.x 14653319

[4] Qin P Munang’andu HM Xu C Xie J. *Megalocytivirus* and other members of the family iridoviridae in finfish: A review of the etiology, epidemiology, diagnosis, prevention and control. Viruses. (2023) 15:1359. doi: 10.3390/v15061359 37376659 PMC10305399

[5] Kurita J Nakajima K. Megalocytiviruses. Viruses. (2012) 4:521–38. doi: 10.3390/v4040521 PMC3347321 22590684

[6] Kwon WJ Choi JC Hong S Kim YC Jeong MG Min JG. Development of a high-dose vaccine formulation for prevention of *megalocytivirus* infection in rock bream (*Oplegnathus fasciatus*). Vaccine. (2020) 38:8107–15. doi: 10.1016/j.vaccine.2020.11.001 33189430

[7] Zhang Q Gui J-F. Virus genomes and virus-host interactions in aquaculture animals. Sci China Life Sci. (2015) 58:156–69. doi: 10.1007/s11427-015-4802-y 25591452

[8] Guo S Wen Z Zhang X Li F Cui H Lin X. Characterization of five caspase genes and their transcriptional changes in response to exogenous iridescent virus challenge in the whiteleg shrimp (*Litopenaeus vannamei*). Aquaculture. (2021) 534:736192. doi: 10.1016/j.aquaculture.2020.736192

[9] Buenrostro JD Giresi PG Zaba LC Chang HY Greenleaf WJ. Transposition of native chromatin for fast and sensitive epigenomic profiling of open chromatin, DNA-binding proteins and nucleosome position. Nat Methods. (2013) 10:1213–8. doi: 10.1038/nmeth.2688 PMC3959825 24097267

[10] Klemm SL Shipony Z Greenleaf WJ. Chromatin accessibility and the regulatory epigenome. Nat Rev Genet. (2019) 20:207–20. doi: 10.1038/s41576-018-0089-8 30675018

[11] Kong X Wei G Chen N Zhao S Shen Y Zhang J. Dynamic chromatin accessibility profiling reveals changes in host genome organization in response to baculovirus infection. PloS Pathog. (2020) 16:e1008633. doi: 10.1371/journal.ppat.1008633 32511266 PMC7326278

[12] Hu S Yang S Lu Y Deng Y Li L Zhu J. Dynamics of the transcriptome and accessible chromatin landscapes during early goose ovarian development. Front Cell Dev Biol. (2020) 8:196. doi: 10.3389/fcell.2020.00196 32309280 PMC7145905

[13] Sun Y Miao N Sun T. Detect accessible chromatin using ATAC-sequencing, from principle to applications. Hereditas. (2019) 156:29. doi: 10.1186/s41065-019-0105-9 31427911 PMC6696680

[14] Pritykin Y van der Veeken J Pine AR Zhong Y Sahin M Mazutis L. A unified atlas of CD8 T cell dysfunctional states in cancer and infection. Mol Cell. (2021) 81:2477–2493.e10. doi: 10.1016/j.molcel.2021.03.045 33891860 PMC8454502

[15] Li M Li J Zhang Y Zhai Y Chen Y Lin L. Integrated ATAC-seq and RNA-seq data analysis identifies transcription factors related to rice stripe virus infection in *Oryza sativa* . Mol Plant Pathol. (2024) 25:e13446. doi: 10.1111/mpp.13446 38502176 PMC10950023

[16] Wu C Zhao X Babu VS Yuan G Wang W Su J. Distribution of mannose receptor in blunt snout bream (*Megalobrama amblycephala*) during the embryonic development and its immune response to the challenge of *Aeromonas hydrophila* . Fish Shellfish Immunol. (2018) 78:52–9. doi: 10.1016/j.fsi.2018.03.049 29627477

[17] Wu C Dai Y Yuan G Su J Liu X. Immunomodulatory effects and induction of apoptosis by different molecular weight chitosan oligosaccharides in head kidney macrophages from blunt snout bream (*Megalobrama amblycephala*). Front Immunol. (2019) 10:869. doi: 10.3389/fimmu.2019.00869 31156612 PMC6530513

[18] Ma Z-Y Liang J-X Li W-S Sun Y Wu C-S Hu Y-Z. Complement C3a enhances the phagocytic activity of B cells through C3aR in a fish. Front Immunol. (2022) 13:873982. doi: 10.3389/fimmu.2022.873982 35386704 PMC8977587

[19] Hu R-D Zhang W Li L Zuo Z-Q Ma M Ma J-F. Chromatin accessibility analysis identifies the transcription factor ETV5 as a suppressor of adipose tissue macrophage activation in obesity. Cell Death Dis. (2021) 12:1–11. doi: 10.1038/s41419-021-04308-0 34716308 PMC8556336

[20] Cao L Luo Y Guo X Liu S Li S Li J. SAFA facilitates chromatin opening of immune genes through interacting with anti-viral host RNAs. PloS Pathog. (2022) 18:e1010599. doi: 10.1371/journal.ppat.1010599 35658050 PMC9200321

[21] Cao L Liu S Li Y Yang G Luo Y Li S. The nuclear matrix protein SAFA surveils viral RNA and facilitates immunity by activating antiviral enhancers and super-enhancers. Cell Host Microbe. (2019) 26:369–384.e8. doi: 10.1016/j.chom.2019.08.010 31513772

[22] Wang XH Wang KR Nie P Chen XH Ao JQ. Establishment and characterization of a head kidney cell line from large yellow croaker *Pseudosciaena crocea* . J Fish Biol. (2014) 84:1551–61. doi: 10.1111/jfb.12386 24773544

[23] Corces MR Trevino AE Hamilton EG Greenside PG Sinnott-Armstrong NA Vesuna S. An improved ATAC-seq protocol reduces background and enables interrogation of frozen tissues. Nat Methods. (2017) 14:959–62. doi: 10.1038/nmeth.4396 PMC5623106 28846090

[24] Chuan J Zhou A Hale LR He M Li X. Atria: an ultra-fast and accurate trimmer for adapter and quality trimming. GigaByte. (2021) 2021:gigabyte31. doi: 10.46471/gigabyte.31 36967729 PMC10038132

[25] Brown J Pirrung M McCue LA. FQC Dashboard: integrates FastQC results into a web-based, interactive, and extensible FASTQ quality control tool. Bioinformatics. (2017) 33:3137–9. doi: 10.1093/bioinformatics/btx373 PMC5870778 28605449

[26] Langmead B Wilks C Antonescu V Charles R. Scaling read aligners to hundreds of threads on general-purpose processors. Bioinformatics. (2019) 35:421–32. doi: 10.1093/bioinformatics/bty648 PMC6361242 30020410

[27] Tarasov A Vilella AJ Cuppen E Nijman IJ Prins P. Sambamba: fast processing of NGS alignment formats. Bioinformatics. (2015) 31:2032–4. doi: 10.1093/bioinformatics/btv098 PMC4765878 25697820

[28] Ramírez F Ryan DP Grüning B Bhardwaj V Kilpert F Richter AS. deepTools2: a next generation web server for deep-sequencing data analysis. Nucleic Acids Res. (2016) 44:W160–5. doi: 10.1093/nar/gkw257 PMC4987876 27079975

[29] Liu T. Use model-based analysis of chIP-seq (MACS) to analyze short reads generated by sequencing protein–DNA interactions in embryonic stem cells. In: Kidder BL, editor. Stem cell transcriptional networks. Methods in molecular biology. Springer New York, New York, NY (2014). p. 81–95. doi: 10.1007/978-1-4939-0512-6_4 24743991

[30] Quinlan AR Hall IM. BEDTools: a flexible suite of utilities for comparing genomic features. Bioinformatics. (2010) 26:841–2. doi: 10.1093/bioinformatics/btq033 PMC2832824 20110278

[31] Yu G Wang L-G He Q-Y. ChIPseeker: an R/Bioconductor package for ChIP peak annotation, comparison and visualization. Bioinformatics. (2015) 31:2382–3. doi: 10.1093/bioinformatics/btv145 25765347

[32] Heinz S Benner C Spann N Bertolino E Lin YC Laslo P. Simple combinations of lineage-determining transcription factors prime cis-regulatory elements required for macrophage and B cell identities. Mol Cell. (2010) 38:576–89. doi: 10.1016/j.molcel.2010.05.004 PMC2898526 20513432

[33] Ross-Innes CS Stark R Teschendorff AE Holmes KA Ali HR Dunning MJ. Differential oestrogen receptor binding is associated with clinical outcome in breast cancer. Nature. (2012) 481:389–93. doi: 10.1038/nature10730 PMC3272464 22217937

[34] Robinson MD McCarthy DJ Smyth GK. edgeR: a Bioconductor package for differential expression analysis of digital gene expression data. Bioinformatics. (2010) 26:139–40. doi: 10.1093/bioinformatics/btp616 PMC2796818 19910308

[35] Dobin A Davis CA Schlesinger F Drenkow J Zaleski C Jha S. STAR: ultrafast universal RNA-seq aligner. Bioinformatics. (2013) 29:15–21. doi: 10.1093/bioinformatics/bts635 23104886 PMC3530905

[36] Pertea M Pertea GM Antonescu CM Chang T-C Mendell JT Salzberg SL. StringTie enables improved reconstruction of a transcriptome from RNA-seq reads. Nat Biotechnol. (2015) 33:290–5. doi: 10.1038/nbt.3122 PMC4643835 25690850

[37] Liao Y Smyth GK Shi W. featureCounts: an efficient general purpose program for assigning sequence reads to genomic features. Bioinformatics. (2014) 30:923–30. doi: 10.1093/bioinformatics/btt656 24227677

[38] Love MI Huber W Anders S. Moderated estimation of fold change and dispersion for RNA-seq data with DESeq2. Genome Biol. (2014) 15:550. doi: 10.1186/s13059-014-0550-8 25516281 PMC4302049

[39] Yu G Wang L-G Han Y He Q-Y. clusterProfiler: an R package for comparing biological themes among gene clusters. Omics J Integr Biol. (2012) 16:284–7. doi: 10.1089/omi.2011.0118 PMC3339379 22455463

[40] Kancherla J Yang Y Chae H Corrada Bravo H. Epiviz File Server: Query, transform and interactively explore data from indexed genomic files. Bioinformatics. (2020) 36:4682–90. doi: 10.1093/bioinformatics/btaa591 PMC7695125 32618995

[41] Yu G Wang L-G Yan G-R He Q-Y. DOSE: an R/Bioconductor package for disease ontology semantic and enrichment analysis. Bioinformatics. (2015) 31:608–9. doi: 10.1093/bioinformatics/btu684 25677125

[42] Martin M. Cutadapt removes adapter sequences from high-throughput sequencing reads. EMBnet.journal. (2011) 17:10. doi: 10.14806/ej.17.1.200

[43] Zhang Q Cheng T Jin S Guo Y Wu Y Liu D. Genome-wide open chromatin regions and their effects on the regulation of silk protein genes in Bombyx mori. Sci Rep. (2017) 7:12919. doi: 10.1038/s41598-017-13186-6 29018289 PMC5635003

[44] Kim A Yoon D Lim Y Roh HJ Kim S Park C-I. Co-expression network analysis of spleen transcriptome in rock bream (*Oplegnathus fasciatus*) naturally infected with rock bream iridovirus (RBIV). Int J Mol Sci. (2020) 21:1707. doi: 10.3390/ijms21051707 32131541 PMC7084886

[45] Bentsen M Goymann P Schultheis H Klee K Petrova A Wiegandt R. ATAC-seq footprinting unravels kinetics of transcription factor binding during zygotic genome activation. Nat Commun. (2020) 11:4267. doi: 10.1038/s41467-020-18035-1 32848148 PMC7449963

[46] Song D Lian Y Zhang L. The potential of activator protein 1 (AP-1) in cancer targeted therapy. Front Immunol. (2023) 14:1224892. doi: 10.3389/fimmu.2023.1224892 37483616 PMC10361657

[47] Qiao Y He H Jonsson P Sinha I Zhao C Dahlman-Wright K. AP-1 is a key regulator of proinflammatory cytokine TNFα-mediated triple-negative breast cancer progression. J Biol Chem. (2016) 291:5068–79. doi: 10.1074/jbc.M115.702571 PMC4777842 26792858

[48] Biddie SC John S Sabo PJ Thurman RE Johnson TA Schiltz RL. Transcription factor AP1 potentiates chromatin accessibility and glucocorticoid receptor binding. Mol Cell. (2011) 43:145–55. doi: 10.1016/j.molcel.2011.06.016 PMC3138120 21726817

[49] Zhao X-F Pan H-F Yuan H Zhang W-H Li X-P Wang G-H. Increased serum interleukin 17 in patients with systemic lupus erythematosus. Mol Biol Rep. (2010) 37:81–5. doi: 10.1007/s11033-009-9533-3 19347604

[50] Kyriakis JM. Activation of the AP-1 transcription factor by inflammatory cytokines of the TNF family. Gene Expr. (1999) 7:217. Available at: https://pmc.ncbi.nlm.nih.gov/articles/PMC6174675/. 10440223 PMC6174675

[51] Monje P Hernández-Losa J Lyons RJ Castellone MD Gutkind JS. Regulation of the transcriptional activity of c-fos by ERK: A NOVEL ROLE FOR THE PROLYL ISOMERASE PIN1*. J Biol Chem. (2005) 280:35081–4. doi: 10.1074/jbc.C500353200 16123044

[52] Roy S Guler R Parihar SP Schmeier S Kaczkowski B Nishimura H. Batf2/Irf1 induces inflammatory responses in classically activated macrophages, lipopolysaccharides, and mycobacterial infection. J Immunol Baltim Md 1950. (2015) 194:6035–44. doi: 10.4049/jimmunol.1402521 25957166

[53] Guo Z Zhu Y Xiao H Dai R Yang W Xue W. Integration of ATAC-seq and RNA-seq identifies MX1-mediated AP-1 transcriptional regulation as a therapeutic target for Down syndrome. Biol Res. (2023) 56:67. doi: 10.1186/s40659-023-00474-x 38066591 PMC10709892

[54] Atsaves V Leventaki V Rassidakis GZ Claret FX. AP-1 transcription factors as regulators of immune responses in cancer. Cancers. (2019) 11:1037. doi: 10.3390/cancers11071037 31340499 PMC6678392

[55] Yu X Wang Y Song Y Gao X Deng H. AP-1 is a regulatory transcription factor of inflammaging in the murine kidney and liver. Aging Cell. (2023) 22:e13858. doi: 10.1111/acel.13858 37154113 PMC10352569

