# Supplementary material for: Chromatin accessibility and differentially expressed genes profiling in large yellow croaker (Larimichthys crocea) head kidney cells following iridovirus infection
Source: Front Immunol. 2025 Jan 30;16:1513966. doi: 10.3389/fimmu.2025.1513966 (PMC11821590; doi:10.3389/fimmu.2025.1513966)
Supplement: Supplementary file 1 [file DataSheet1.docx]

**Supplementary Figure 1**

**
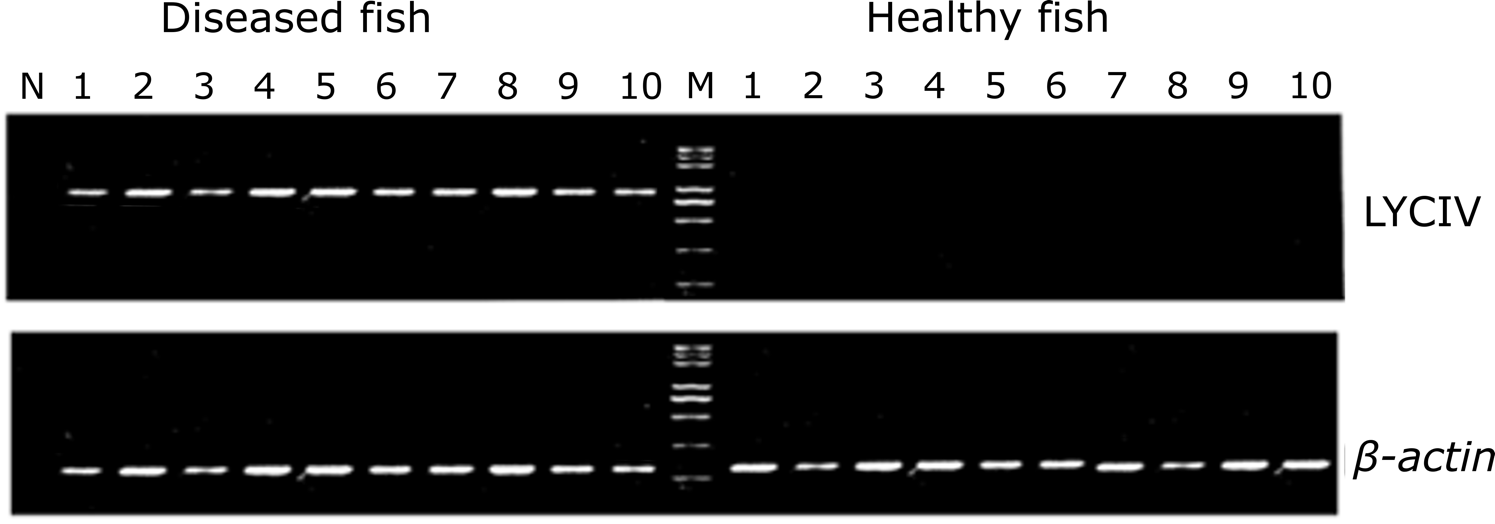
**

**Figure S1. Agarose gel electrophoresis of the PCR products for LYCIV viral load determination. *β-actin* gene was used as an internal control.**

**Supplementary Figure 2**
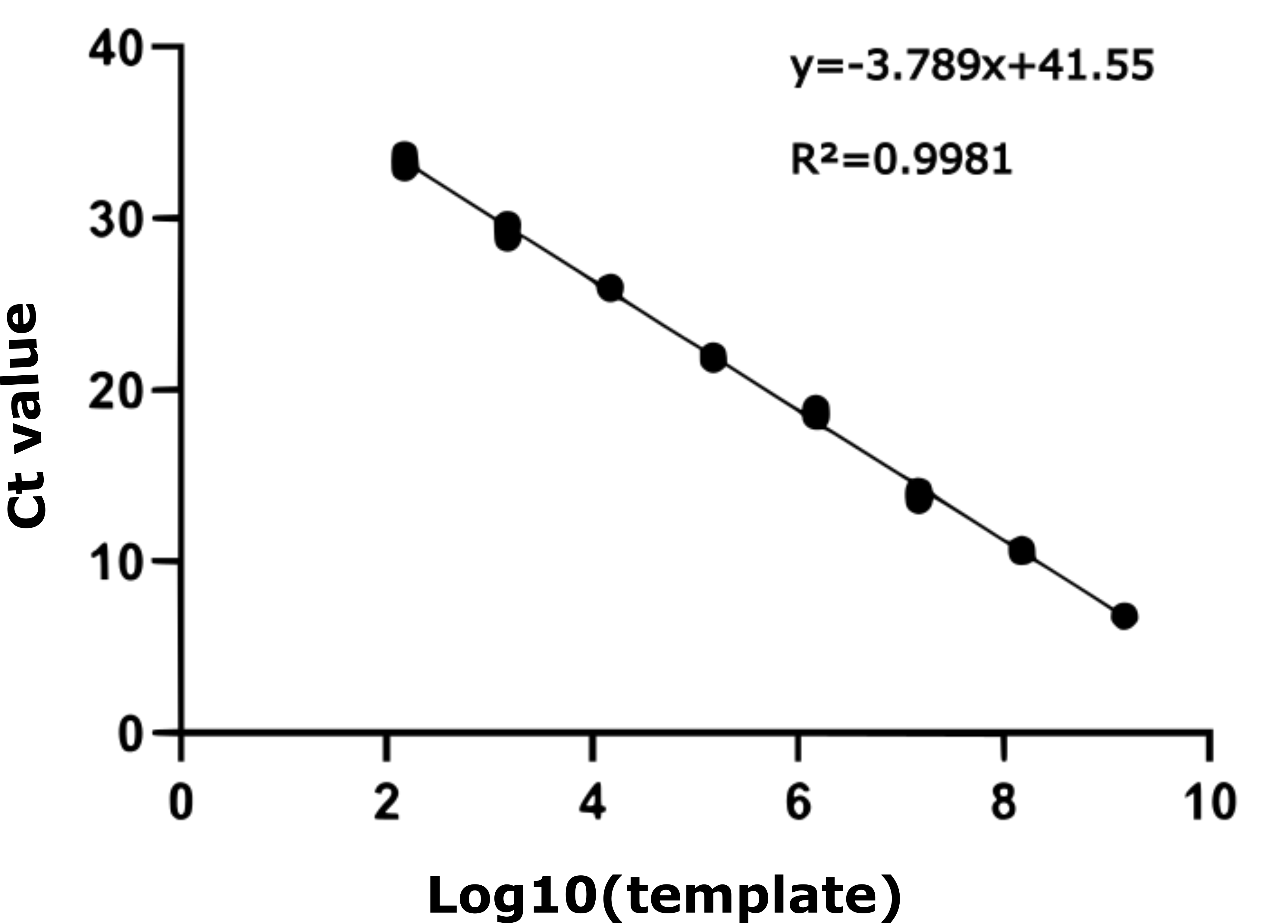


**Figure S2. Standard curve for detecting LYCIV copy numbers.**
